# Redefining disease in the age of blood-based biomarkers

**DOI:** 10.3389/fsoc.2025.1533429

**Published:** 2025-02-28

**Authors:** Naveen K. Reddy

**Affiliations:** Department of Neurology, University of California, San Francisco, San Francisco, CA, United States

**Keywords:** Foucault, Ivan Illich, Alzheimer's disease, amyloid, disease mongering

## Abstract

This article explores the sociological and ethical implications of redefining disease in the era of advanced diagnostic technologies, with a focus on blood-based biomarkers. Drawing from Foucault's concept of medicalization and Illich's critique of disease mongering, it highlights how diagnostic expansions, driven by corporate and institutional influences, are reshaping the boundaries of health and disease. Advances such as blood assays for Alzheimer's and Parkinson's diseases, liquid biopsies in oncology, and biomarkers for depression and diabetes, while promising, raise concerns about premature diagnoses and overtreatment. The influence of pharmaceutical and insurance industries on diagnostic criteria, as seen in the ICD updates, underscores the need to address conflicts of interest and regulatory gaps. Case studies on Alzheimer's and Parkinson's reveal how these changes could benefit stakeholders at the expense of patient welfare. The article calls for ethical oversight, stricter regulation, and research into the population-level efficacy of diagnostic and treatment protocols.

## Introduction

“The gaze that the doctor casts on the patient is not that of a singular individual. It is a gaze that is structured by a whole set of medical knowledge. The patient's body is no longer the site of a personal experience but the field of an objectified experience of disease.”—Michel Foucault, *The Birth of the Clinic*

With the publication of Foucault's *Birth of the Clinic*, a whole field of medical sociology was born with its main goal to understand how the State, by institutionalizing the field of medicine, exerts power over the human body. In turn, many sociologists over the years, most notably Ivan Illich, have shown how normal variations in human behaviors can be deemed pathological and thereby made open to medicalization (Illich, 1982). This phenomenon—termed “disease mongering”—was mostly confined to the field of psychiatry, but over the past three decades, we have seen it enter nearly every field of medicine. Mostly due to advances in the field of biological chemistry with the development of highly sensitive blood-based biomarkers (Barthélemy et al., 2024). While these advances are groundbreaking in their ability to detect minuscule biological data, they come with a whole host of ethical and sociological consequences. Namely, how will the power of these advanced technologies be wielded by the dominant institutions within our society—namely the medical-industrial complex? And in turn, what structures should be put in place to prevent the exploitation of patients?

## The influence of industry on diagnostic criteria

How we define disease fundamentally shapes how we treat it. While this may seem straightforward, what often goes unnoticed is the frequency with which these definitions change. For example, recent adjustments in hypertension criteria lowered the threshold for systolic blood pressure from >140/90 to >130/90 (Ioannidis, 2018). Though the shift appears small, it has resulted in millions of new hypertension diagnoses, prompting more aggressive prescribing of anti-hypertensives and reshaping clinical practice (Sekkarie and Loustalot, 2024).

Historically, revisions to disease definitions were rooted in rigorous research and designed to improve patient care. After being validated by scientific consensus, these changes were adopted by professional bodies composed of impartial physicians and researchers. They were then translated into diagnostic codes by institutions like the CMS and WHO (World Health Organization, 2024). However, in recent years, this process has become increasingly influenced by external forces—including biopharmaceutical corporations, insurance companies, and other stakeholders whose interests often conflict with purely medical considerations (Wouters, 2020).

Today, we're seeing the consequences of this influence reflected in the constant expansion of the ICD (International Classification of Diseases) diagnostic manual (World Health Organization, 2024). Between ICD-9, released in 1974, and ICD-10, released in 1994, the number of diagnostic codes expanded from 13,000 to 68,000. And between 2016 and 2024, ICD-10 underwent further revisions, adding over 1,000 new codes each year, including 395 in 2023 alone (Wolters Kluwer, 2023). While many of these revisions are due to our expanding medical knowledge, there have been numerous cases of industry-influenced diagnostic criteria changes e.g., in the management of osteoporosis, male pattern balding, irritable bowel syndrome, and ADD. In turn, the CMS and WHO are increasingly being pulled toward promoting the interests of the medical-industrial complex over their responsibility to the public welfare.

## Alzheimer's and Parkinson's disease: emerging case studies in diagnostic expansion

A concrete example is currently unfolding in the field of Alzheimer's disease with the advent of a blood-based assay able to detect the amyloid plaques and tau tangles that define the disease with high diagnostic accuracy (Barthélemy et al., 2024). When combined with traditional cognitive testing, they offer an effective means of diagnosing Alzheimer's in symptomatic patients. The key term here is symptomatic patients. Drug manufacturers are now proposing to diagnose individuals with “Stage 1” Alzheimer's based on biomarker positivity alone, even when no cognitive symptoms are present (Peterson, 2024). In short, these companies are attempting to diagnose Alzheimer's disease in patients who don't have memory problems. To truly understand the implications of this, we need to remember that over the past year, several anti-amyloid agents that attempt to treat Alzheimer's disease have entered the market (lecanemab, donanemab). In turn, these therapies if given to both symptomatic and asymptomatic patients, will be astoundingly lucrative for drug manufacturers. It is no coincidence then that the FDA approval of these anti-amyloid agents has led to an acceleration in the development of blood-based biomarkers and in turn, an attempt by the industry to revise diagnostic criteria to facilitate the widespread use of these drugs without good evidence for doing so.

In Parkinson's disease, a similar trend is emerging with synuclein seed amplification assays, which detect alpha-synuclein, the pathological protein associated with Parkinson's (Oftedal et al., 2023). Studies are underway to detect misfolded proteins in asymptomatic individuals, mirroring the trajectory seen in Alzheimer's research. While a disease-modifying treatment for Parkinson's hasn't come to market just yet, once it does, the definition of Parkinson's disease and possibly all synucleinopathies will likely be expanded to catch asymptomatic individuals—just as it's being proposed for Alzheimer's.

## Beyond neurology

This phenomenon isn't just isolated to neurological diseases. In oncology, liquid biopsies, which detect circulating tumor DNA, may expand cancer definitions to preclinical stages that might never progress to symptomatic disease (Ignatiadis et al., 2021). In psychiatry, blood tests for inflammation markers linked to depression could pathologize normal emotional responses (Strawbridge et al., 2017). Even in conditions like diabetes, advanced biomarkers for insulin resistance might classify individuals without symptoms as pre-diabetic, pushing them toward the use of expensive GLP-1 agonists (Cronjé et al., 2023).

## Navigating the ethics of advanced diagnostics

We have entered a new era of advanced diagnostic medical technology with the advent of such powerful blood-based assays. Drawing on Foucault's work, we can see how advanced diagnostics not only shift the boundaries of disease but also restructure power dynamics, placing certain incentives above patient care (Foucault, 2020).

Just as is the case for any advanced technology, significant harm can occur if these tools are misused. To prevent such exploitation of these diagnostic advancements, it is essential that we reinforce our regulations around conflicts of interest and work toward a clear separation of industry from the diagnostic process. This would entail that our professional societies and governmental institutions, such as CMS and WHO, search for and remove players with strong corporate interests within their ranks. This would entail stronger penalties for pharmaceutical manufacturers who are caught attempting to unduly influence the development of diagnostic criteria. And it would entail we invest in research that attempts to determine if our current diagnostic and treatment algorithms are truly efficacious at a population level. These powerful medical technologies have the potential to help so many patients, but at the same time, we must demand that they're provided safely and equitably.

As Foucault wrote, “The problem is not how to get rid of power, but how to use it… The task, therefore, is not to seek its elimination, but to question its deployment, and to ensure that it does not operate in ways that reinforce inequality or harm.”
